# Exploring the umbilical and vaginal port during minimally invasive surgery

**DOI:** 10.4274/jtgga.2017.0046

**Published:** 2017-09-01

**Authors:** Andrea Tinelli, Daniel A. Tsin, Antonello Forgione, Ricardo Zorron, Giovanni Dapri, Antonio Malvasi, Tahar Benhidjeb, Radmila Sparic, Farr Nezhat

**Affiliations:** 1 Department of Gynecology and Obstetrics, Division of Experimental Endoscopic Surgery, Imaging, Minimally Invasive Therapy and Technology, Vito Fazzi Hospital, Lecce, Italy; 2 Laboratory of Human Physiology, Department of Applied Mathematics, Moscow Institute of Physics and Technology (MIPT), State University, Moscow, Russia; 3 The Mount Sinai Hospital of Queens, Long Island City, New York, USA; 4 Advanced International Mini-Invasive Surgery Academy, Milan, Italy; 5 Center for Innovative Surgery (ZIC), Center for Bariatric and Metabolic Surgery, Department of General, Visceral and Transplant Surgery, Campus Virchow Klinikum and Department of General, Visceral, Vascular and Thoracic Surgery, Campus Mitte, Charité-Universitätsmedizin, Berlin, Germany; 6 Department of Gastrointestinal Surgery, European School of Laparoscopic Surgery, Saint-Pierre University Hospital, Brussels, Belgium; 7 Department of Obstetrics and Gynecology, Santa Maria Hospital, GVM Care&Research, Bari, Italy; 8 Consultant, German Board-Surgery; Chairman, Department of Surgery; Chief, General Surgery Danat Al Emarat Hospital, UAE; 9 Clinic of Gynecology and Obstetrics, Clinical Center of Serbia, University of Belgrade School of Medicine, Belgrade, Serbia; 10 Department of Obstetrics, Gynecology and Reproductive Medicine, State University of New York at Stony Brook School of Medicine, Stony Brook, NY; Department of Obstetrics and Gynecology, Winthrop University Hospital, Mineola, NY, USA; 11 Department of Obstetrics, Gynecology, and Reproductive Science, Icahn School of Medicine at Mount Sinai, New York, NY, USA

**Keywords:** Single port laparoscopy, culdolaparoscopy, natural endoscopic surgery, colpotomy, postoperative pain

## Abstract

This article focuses on the anatomy, literature, and our own experiences in an effort to assist in the decision-making process of choosing between an umbilical or vaginal port. Umbilical access is more familiar to general surgeons; it is thicker than the transvaginal entry, and has more nerve endings and sensory innervations. This combination increases tissue damage and pain in the umbilical port site. The vaginal route requires prophylactic antibiotics, a Foley catheter, and a period of postoperative sexual abstinence. Removal of large specimens is a challenge in traditional laparoscopy. Recently, there has been increased interest in going beyond traditional laparoscopy by using the navel in single-incision and port-reduction techniques. The benefits for removal of surgical specimens by colpotomy are not new. There is increasing interest in techniques that use vaginotomy in multifunctional ways, as described under the names of culdolaparoscopy, minilaparoscopy-assisted natural orifice surgery, and natural orifice transluminal endoscopic surgery. Both the navel and the transvaginal accesses are safe and convenient to use in the hands of experienced laparoscopic surgeons. The umbilical site has been successfully used in laparoscopy as an entry and extraction port. Vaginal entry and extraction is associated with a lower risk of incisional hernias, less postoperative pain, and excellent cosmetic results.

## INTRODUCTION

Laparotomy was replaced by laparoscopy for many procedures. With advances in techniques, tools and materials, new methods of minilaparoscopy and single-access surgery (SAS) for umbilical, vaginal or other natural orifices evolved. Abdominal extraction of surgical specimens is not possible when 3.5 mm or smaller ports are the only ones placed in the abdomen, whereas some surgical specimens could be removed via traditional 10-mm or 12-mm laparoscopic umbilical ports. For large specimens, the solutions include extension of the umbilical port or a secondary port, minilaparotomy, morcellation, and colpotomy. The greatest advantage of extraction through an enlarged umbilical incision is the possibility of performing laparoscopic procedures with secondary ports of 5 mm, minilaparoscopy instruments or percutaneous needles. Another problem faced when large specimens have to be extracted through secondary ancillary ports is damage to the inferior epigastric artery during port enlarging (1). The inferior epigastric artery is a collateral branch of the external iliac artery (EIA), which usually arises from the EIA, 6.2 cm above the inguinal ligament. Nevertheless, the EIA can arise from the femoral artery below the inguinal ligament, from the profunda femoral artery, and even as a common trunk with the circumflex iliac artery. Lesions incurred during enlargement of lateral ancillary ports can lead to a severe hemorrhage with potentially serious complications.

SAS is usually performed via a unique navel incision and multichannel ports. Operative laparoscopes with working channels were also used with the aid of percutaneous needle assistance. Port size extension increases tissue damage and can increase the chances of injury to inferior epigastric vessels, port-site hernia (PSH), postoperative pain, and poor cosmetic results (2).

Colpotomy has been published under different names such as vaginotomy, vaginal celiotomy, or culdotomy and circular colpotomy during vaginal hysterectomy. It has been used during surgery and for extraction. Some laparoscopists used colpotomy as an extraction option rather than increasing or making an additional abdominal incision.

Culdolaparoscopy (3), minilaparoscopy-assisted natural orifice surgery (MANOS) (4), and natural orifice transluminal endoscopic surgery (NOTES) use multifunctional vaginal access for insufflation, viewing, surgery, and extraction (5, 6, 7). In this paper, we attempt to compare umbilical and vaginal extraction, specifically when large specimen are retrieved, focusing on anatomic differences based on the literature and our own experience.

## VAGINAL ACCESS ANATOMY

Most general surgeons rarely use a vaginal port for access or extraction of samples. Therefore, this section serves as a refresher for non-gynecologic surgeons to consider vaginal extraction or use of the transvaginal approach in culdolaparoscopy, MANOS or NOTES.

The vagina is a fibro-muscular structure, S-shaped, between 6 and 12 cm long, and 3 to 4 mm thick, being longer in the rear wall (8). The cephalic or upper section of the vagina widens to form a pouch around the cervix, which is known as the fornix. The fornix can be separated in the anterior, posterior and, lateral fornixes. The lateral fornixes are topographic in relation with the endo pelvic fascia and the base of the broad ligaments. The vaginal wall of the posterior fornix is in contact with the peritoneum of the pouch of Douglas (posterior cul-de-sac). The posterior vaginal wall is divided into 3 sections; the upper is the fornix, as previously described. The middle portion is attached to the rectum vaginal septum. The lower portion is attached to the rectovaginal septum and the fibromuscular nucleus of the perineal body. We have not used the anterior fornix colpotomy in these surgeries even though we use the circular colpotomy during vaginal hysterectomies (8, 9). Colpotomy has been used for specimen extraction by laparoscopists in general surgery, gynecology, and urology (10).

Harlaar et al. (11) measured the cavity of the posterior cul-de-sac in ten cadavers. They used latex to make a mold of the posterior cul-de-sac. The mean diameter was 2.6 cm (±0.5 cm) with a range of 2.0-3.4 cm (11). However, these measurements are limited to those of a cadaver. In a live female, the measurements are significantly different when performing laparoscopy (12). We use a uterine manipulator to bring the uterus in an anterior and cephalic direction. This maneuver opens the pouch of Douglas under an endoscopic view. It broadens the posterior “cul-de sac” by several centimeters and allows the placement of a vaginal port, as well as removal of the appendix, gallbladder, ovarian cysts, uterine fibroids, and other types of specimens. When the removed sample is large, suction, drainage, and collapse of a cyst or hollow viscous is performed. For a large solid specimen, hemisecting or morcellating is needed to reduce the size in order to avoid damage by overstretching the anatomically related structures (9).

## UMBILICAL ACCESS ANATOMY

The introduction of SAS has enabled the navel to be reconsidered as an embryonic natural orifice that can be used as the main access for general laparoscopy, offering the advantage of surgery without scars. Therefore, we review these structures in order to prevent complications (13).

Typically, the average navel is 8.2 mm in thickness when measured in the shortest length between the skin and peritoneum. It is slight thicker in women than in men (14). The navel is located at the level of the highest point of the iliac crest, the third to fourth lumbar disc, and equidistant between the tip of the xiphoid process and the top of the pubic symphysis. The navel is located slightly depressed and down in the middle of the depth of the abdominal wall, facing the body at L4. The skin bottom of the umbilical depression has a protrusion, which is in a bun shape form, the umbilical tuber, which is separated from the peripheral skin by a circular groove. In the center of the tuber is the scar of the umbilical cord. The position of the navel changes during pregnancy or obesity (15). The subcutaneous layer consists of fatty tissue and small arteries, veins and nerves, and is thicker in the obese. The deeper layer of the navel is composed of fibrous tissue covered by sub peritoneal tissue were the umbilical artery and veins run. The umbilical ring is embedded in the middle linea Alba. The navel ring includes the insertion of the urachus, remnants of the umbilical arteries caudally, and the Teres ligament cephalically, all of which are covered by the parietal peritoneum (16).

## DIFFERENCES IN UMBILICAL AND VAGINAL INNERVATIONS

To better understand many of the differences between the umbilicus and vagina as an inlet for surgery, we review the innervations of these organs (17). The umbilical area is very sensitive when compared with others areas in the abdomen. In a study of innervations of the umbilical skin, authors are quoted to have found epidermal and dermal numerous tactile cells and corpuscles, and abundant end-bulblike genital structures. Nerve bundles were observed together with bulbous and lamellar corpuscles with abundant nerve endings both in the papillary and reticular dermis (18).

The vagina receives visceral and somatic nerves. The innervations arrive via the lower hypogastric plexus and the internal pudendal nerve, forming a plexus around the vagina (16, 19). The posterior vaginal fornix area has substantially less sensory innervation than the navel area.

Griebling et al. (20) described a neural-rich submucosal plexus within the region of the vagina, with a small quantity of intraepithelial axons. The sympathetic and parasympathetic nerves are smaller and have even less presence of sensory nerves (20). Their study showed that innervations changed under the influences of estrogen (20, 21).

Postmenopausal women often experience some level of atrophy due to lower estrogen levels, which produce changes in innervations. Treatments with estrogen replacement should improve this condition (22, 23, 24, 25, 26, 27).

## INVESTIGATIONS ON VAGINAL PORT AND SEXUAL FUNCTION

Regarding sexual function after posterior colpotomy, Butticè et al. (28) evaluated 71 patients after hybrid transvaginal (NOTES) nephrectomy. Sexual function was evaluated using the Female Sexual Function Index (FSFI) questionnaire the day prior to the operation and 3 months after. The authors showed that the complication risk increased significantly with increasing tumor size. Among the whole cohort, even the FSFI score differences were small; there was a statistically significant decrease in the postoperative period in all domains, except sexual satisfaction. In fact, the patients reported unaltered sexual function after surgery and satisfaction with the result when asked directly. In subgroup analyses, in nulliparous patients (n=60), arousal, sexual desire, orgasm, and satisfaction domains had no significant differences in pre- and postoperative periods. Accordingly, the authors strongly supported the use of hybrid transvaginal NOTES nephrectomy for large renal tumors, especially in nulliparous patients, for unaltered sexual satisfaction (28).

Nevertheless, Vitale et al. (29) also studied quality of life (QoL) and sexual function changes of 20 women with third- and fourth-degree cystocele (according to the Baden-Walker classification), treated using biocompatible porcine dermis graft. The Short Form-36 questionnaire to assess QoL was administrated at baseline and 12 months after surgical treatment. The Pelvic Organ Prolapse/Urinary Incontinence Sexual Questionnaire (PISQ-12), which measures changes of sexual behavior, was used at baseline and 12 months after surgical treatment. Each woman underwent translabial color Doppler ultrasonography to measure clitoral blood flow before and 12 months after surgical treatment. In the results, the authors showed that use of biocompatible porcine dermis grafts to treat severe cystocele considerably improved QoL and sexual function, and did not influence clitoral blood flow. Thus, based on the authors’ experience, vaginal prolapse and urinary incontinence treatment (30) should also provide sexual improvement.

The problem has not yet been completely addressed by surgeons, especially in cases of mass extraction or large specimens. Rolli et al. (31) investigated the feasibility and safety of vaginal myomectomy via posterior colpotomy in a series of 46 consecutive procedures. Thirty-two patients underwent successful vaginal myomectomy under general anesthesia and 12 under loco-regional anesthesia, and the median size of the myomas was 50 mm (range, 16-81 mm). The authors showed the feasibility of the technique, but they did not address sexual function after surgery, or whether any patients achieved pregnancy after myomectomy (31).

## STUDIES ON SURGICAL COMPARISON OF METHODS

Gynecologists have been using transvaginal access to the peritoneal cavity via a posterior colpotomy for more than a century for diagnostic and operative procedures, and specimen extraction (4). However, since the early 1970s, vaginal access was replaced by laparoscopy (32, 33). The reasons why transvaginal access fell out of favor are multifactorial, including advances in laparoscopy, perceived technical difficulty, concerns about patient acceptance, infections, taboos, and misconceptions (34). Recently, with the emergence of MANOS and NOTES peritoneoscopy surgery, the vagina has become the most suitable entry and extraction site to operate on and retrieve several organs in different specialties, such as general surgery, urology and, of course, gynecology (34, 35). Ghezzi et al. (2) reported their experience with large specimen extraction using the navel in 1116 gynecologic laparoscopy procedures. There were no problems with direct surgical extraction of specimens or with endobags. The only complication was a laceration of the epigastric artery. Neither PSH nor metastases occurred. Another study of Ghezzi et al. (33) randomly compared the transumbilical and transvaginal routes for retrieval of adnexal masses in laparoscopy. The results of this study suggest that retrieval of adnexal masses following laparoscopic excision via a posterior vaginotomy causes less postoperative pain than transabdominal specimen extraction through the navel port (35).

Bulian et al. (36) successfully performed hybrid transvaginal cholecystectomies (TVC) using rigid instruments and compared this technique with the traditional laparoscopic technique. In their comparison, there were no postoperative differences in terms of pain and sex in the TVC group, whereas there were two PSHs found in the laparoscopy group. Patients in the TVC group were more satisfied with the results compared with those who underwent traditional laparoscopy. The transvaginal access procedures were mostly performed using prophylactic antibiotics, and required Foley catheter insertion and sexual abstinence. The authors suggested that the postoperative abstinence period varies among different groups, from 14 to 42 days (34).

A recent review (37) reported 11 years’ experience of removing specimens via posterior colpotomy in 230 patients and included 899 cases collected over 17 publications. The data suggested that the removal of transvaginal specimens represented a safe, feasible, and applicable procedure in the field of laparoscopic gynecology. A 13-year experience report of 2431 single-port laparoscopic cholecystectomies performed using a laparoscope with a 6-mm working channel and percutaneous needle assistance documented excellent cosmetic results with a low incidence of PSH (38).

When considering the differences in the two oncologic staging accesses, because laparoscopy should be preferred over laparotomy as stadiation procedure in early-stage and advanced ovarian cancer, it is indisputable to think about the advantages of the umbilical than the vaginal port.

In fact, Rossetti et al. (39) evaluated the feasibility, safety, and effectiveness of laparoendoscopic single-site surgery (LESS) for the assessment of peritoneal carcinomatosis resectability in 55 patients with advanced-stage ovarian cancer (AOC). The authors reviewed the medical records of patients with AOC who underwent LESS for operative examination. Standard cytoreductive laparotomy surgery was performed. The peritoneal cancer score was completed in 49 (94%) patients using LESS; 34/37 (92%) patients who were considered to have resectable disease were effectively optimally debulked. The authors concluded that LESS was a feasible and safe alternative minimally invasive procedure to assess the resectability of patients with AOC (39).

## CONCLUSION

In the era of reduced-port surgery and NOTES, the ideal method to access and perform surgery in the abdominal cavity and extract specimens is under scrutiny. Both navel and vaginal sites are safe and feasible for use by trained laparoscopist surgeons. A review of the literature and our own experience show that the transvaginal route is associated with reduced or no risk of PSH, reduced postoperative pain, faster recovery of common activity, and excellent cosmetic results.

When the vaginal option is not available, the lack of benefits is obvious, specifically if large specimens are to be extracted. In such cases, enlargement of an abdominal port inlet or an additional incision is needed, which increases tissue damage and pain. These maneuvers greatly limit many of the advantages of the minimally invasive approach.
